# Elements of Physiology

**Published:** 1840-01

**Authors:** 


ART. VII.—*Elements of Physiology.* By J. MÜLLER, M.D., Professor of Anatomy and Physiology in the University of Berlin, &c. *Translated from the German, with Notes*, by WILLIAM BALY, M.D. Second Edition, with Corrections and numerous Additions. Part I.—*London*, 1839. 8vo, pp. 471.

WE do not notice this new edition of Dr. Baly's translation of Müller's classical work, with the view of analysing, or even criticising it, but merely for the purpose of recommending it to such of our readers as are still unacquainted with it. It is a production of such uncommon excellence, that, whatever difference of opinion may exist among physiologists respecting certain views and doctrines advanced in it, there can be only one as to its unequalled merits as a system of physiology for the advanced student. And this character applies to it better in its present than in its original form ; inasmuch as the translator has not only preserved all that is of value in the German edition, but has placed several things in a better point of view, and has added many others of no small importance ; while he has contrived, throughout, to achieve the very difficult task of giving a faithful version of the original language, in a perfectly good English style. The appearance of this new edition of the work, at this early period, is in itself a proof of its intrinsic value, and of the merits of the translator and editor. The present Part contains, General Physiology ; the Blood and Circulating System ; the Lymph and Lymphatic System ; Respiration ; Nutrition, Growth, and Reproduction. We recommend the volume, in the strongest terms, to all our readers, and to every student of physiology.

